# Contributions of heavy metal exposure to late‐onset Alzheimer’s disease

**DOI:** 10.1002/alz.091290

**Published:** 2025-01-03

**Authors:** Kevin P Kotredes, Olga Minaeva, Ravi S Pandey, Juliet A Moncaster, Bruce T. Lamb, Gregory W Carter, Lee E Goldstein, Gareth R Howell

**Affiliations:** ^1^ The Jackson Laboratory, Bar Harbor, ME USA; ^2^ Boston University Alzheimer’s Disease Research Center, Boston, MA USA; ^3^ The Jackson Laboratory for Genomic Medicine, Farmington, CT USA; ^4^ Stark Neurosciences Research Institute, Indiana University School of Medicine, Indianapolis, IN USA; ^5^ Boston University Alzheimer's Disease Research Center, Boston, MA USA

## Abstract

**Background:**

Late‐onset Alzheimer’s disease (LOAD) is the leading cause of dementia and a major contributor to increased mortality. Recent human datasets have revealed many LOAD genetic risk factors that are correlated with the degree of AD burden. Further, the complexity and heterogeneity of LOAD appears to be promoted by interactions between genetics and environmental factors such as diet, sedentary behavior, and exposure to toxicants, like lead (Pb), cadmium (Cd), and arsenic (As). While the neurotoxicants‐LOAD association is known, the molecular mechanisms modulated by these gene‐environmental interactions are unknown. Here we test the hypothesis that heavy metal exposure induces cerebrovascular deficits, neuroinflammation, and brain biometal dyshomeostasis which exacerbate AD‐associated brain pathologies in next‐generation mouse models of LOAD. Examination of these gene‐environmental (“exposome”) interactions provides essential insight into the heterogeneity observed in human disease and may uncover potentially modifiable mechanisms that mediate AD pathogenesis.

**Method:**

Young and aged mice from novel polygenic strains expressing LOAD risk alleles (*APOE4, Trem2, APP, Mthfr, Abca7*) were exposed to heavy metal toxicants in drinking water. Toxicants and endogenous biometals were assayed by ICP‐mass spectrometry in the brain, blood, and urine. Transcriptional profiling of brains revealed specific changes in human‐aligned, LOAD‐related gene expression networks indicating mechanisms of disease progression. Neuropathology was evaluated with LOAD‐relevant phenotypes, including amyloid burden, glial activity, and neuron loss.

**Result:**

Neurotoxicants were detected in all tissue samples collected. Pb, Cd, and As accumulated in the brain and altered expression of LOAD‐relevant genes in a toxicant‐specific manner, including a decrease in *Vgf* and an increase in *App*. Reduced *VGF* expression has been observed and reported in all four independent AMP‐AD studies and nominated as a key therapeutic target in each and *APP* encodes amyloid precursor protein (APP) from which the Aβ peptides are generated.

**Conclusion:**

Pb, Cd, and As exposure is common, especially in disadvantaged populations (urban, rural), raising concern about LOAD risk disparities, socioeconomic/racial inequities, and environmental justice. These experiments provide critical feedback related to the impact of the “exposome” in the aging, disease progression, and gene expression of novel preclinical LOAD models. Collectively these data suggest a direct effect of neurotoxicant exposure related to LOAD progression.

